# Migraine before rupture of intracranial aneurysms

**DOI:** 10.1186/1129-2377-14-15

**Published:** 2013-02-20

**Authors:** Elena R Lebedeva, Natalia M Gurary, Vladimir P Sakovich, Jes Olesen

**Affiliations:** 1 Department of Neurology and Neurosurgery, The Urals State Medical Academy, 124 Chkalova str., ap.94, Yekaterinburg, Russia; 2 Department of Neurology, Glostrup Hospital, University of Copenhagen, Copenhagen, Denmark

**Keywords:** Migraine, Headache, Risk factor, Intracranial aneurysm, Unruptured aneurysm, Subarachnoid heamorrhage

## Abstract

**Background:**

Rupture of a saccular intracranial aneurysm (SIA) causes thunderclap headache but it remains unclear whether headache in general and migraine in particular are more prevalent in patients with unruptured SIA.

**Methods:**

In a prospective case–control study 199 consecutive patients with SIA (103 females and 96 males, mean age: 43.2 years) received a semistructured face to face interview focusing on past headaches. All were admitted to hospital mostly because of rupture (177) or for unruptured aneurysm (22). In parallel we interviewed 194 blood donors (86 females, 108 males, mean age: 38.4 years). Diagnoses were made according to the International Headache Society criteria. Aneurysms were diagnosed by conventional cerebral angiography.

**Results:**

During the year before rupture, 124 (62.3%) had one or more types of headache. These headaches included: migraine without aura (MO): 78 (39.2%), migraine with aura (MA): 2 (1%), probable migraine (PM): 4 (2%), tension-type headache (TTH): 39 (19.6%), cluster headache (CH): 2 (1%), posttraumatic headaches (PH): 2 (1%). 1-year prevalence of headaches in controls was 32.5% (63 patients out of 194), they included: TTH: 45 (23.1%), MO: 17(8.8%), PH: 1(0.5%). Only the prevalence of MO was significantly higher in patients with SIA (OR 6.7, 95% CI 3.8-11.9, p < 0.0001).

**Conclusions:**

Unruptured SIA cause a marked increase in the prevalence of migraine without aura but not in the prevalence of other types of headache.

## Background

While the thunderclap headache associated with rupture of saccular intracranial aneurysm (SIA) is well known (ICHD-2) [1-5], it is much less certain if an unruptured SIA can cause headache. SIA of the internal carotid artery can cause severe pain around the eye including radiation to the head because of compression of the third nerve [6-8]. Likewise, giant aneurysms causing compression or distortion of intracranial structures are an accepted cause of headache [9,10]. However, SIA that does not cause compression of a cranial nerve or other structures is not an intuitively obvious cause of headache.

Perhaps for that reason, the question has received little scientific study. In the chapter devoted to aneurysms in a standard reference book on headache [11], it is stated that unruptured SIA may be a cause of headache but the underlying literature references are case stories or uncontrolled studies. Recently the effect of operating unruptured SIA was described [12]. The study showed a reduction of approximately 2/3 of headache prevalence after closure of the aneurysm, but the material was too small to determine the prevalence of headache before treatment and there was no control group [12]. Currently, the importance of cerebral arteries in the pathogenesis of migraine is hotly debated and central mechanisms are generally favored [13]. Any influence of unruptured aneurysms would support a role of cerebral arteries or their innervation in migraine.

On this background it is important to gather reliable information about the prevalence of headache in patients with unruptured SIA and to classify such headaches according to the International Classification of Headache Disorders [14]. To definitively answer these questions, we did a very large, prospective case controlled study interviewing patients shortly after rupture about their headaches before rupture using a purpose-developed standardize semistructured interview that we simultaneously applied to matched blood donors. Our hypothesis was that SIA patients have an increased prevalence of migraine.

## Methods

This prospective case–control study included 199 consecutive patients with saccular intracranial aneurysms (SIA) (103 females and 96 males, mean age: 43.2 years) and 194 control subjects–blood donors at the regional blood transfusion center (86 females, 108 males, mean age: 38.4 years). This was the maximal number that could be achieved. No formal power calculation was done. Clinical characteristics of patients with SIA and controls are presented in Table 1. We consecutively recruited patients with SIA admitted to our regional neurosurgical center and control subject from 2003 to 2006. Aneurysms were verified by conventional cerebral angiography. 36 (18.1%) patients had multiple aneurysms. 22 patients (11%) had unruptured aneurysms. 177 patients (89%) had subarachnoid heamorrhages. 190 patients were operated of whom 177 had clipping and 13 coiling.

**Table 1:** Clinical characteristics of patients with SIA and controls

**Characteristics**	**Patients with SIA (n = 199)**	**Controls (n = 194)**	**P**	**OR (95% CI)**
age range and mean age	14-73 (43.2)	18-59 (38.4)	>0.05	
males	96 (48.2%)	86 (44.3%)	>0.05	
patients with arterial hypertension	115 (57.8%)	18 (9.3%)	<0.0001	13.4 (7.6-23.4)
patients with systemic CTD	125 (62.8%)	23 (11.8%)	<0.0001	12,5 (7,45-21,1)
patients who have relatives with stroke	50 (25.1%)	20 (10.3%)	<0.0001	2.9 (1.7-5.1)
patients who have relatives with SIA	10 (5.0%)	0		
patients who have relatives with headaches	114 (57.3%)	46 (23.7%)	<0.0001	4.3 (2.8-6.6)
smokers	102 (51.2%)	102 (52.6%)	>0.05	
patients with alcohol consumption	69 (34.7%)	77 (39.7%)	>0.05	
patients with increased BMI (>25)	148 (74.4%)	112 (57.7%)	>0.05	
patients with headaches	124 (62.3%)	63 (32.5%)	<0.0001	3.4 (2.3 -5.2)
patients who used analgetics more than 15 times per month	9 (4.5%)	3 (1.5%)	>0.05	

Inclusion criteria for patients with SIA:

1. The patient had at least one saccular intracranial aneurysm confirmed by cerebral angiography

2. The patient lived in Yekaterinburg or in the Urals region.

3. The patient agreed to conduct additional examinations.

Exclusion criteria for patients with SIA

1. The patient had fusiform, traumatic or mycotic aneurysm.

2. Patients who were unable to give a coherent headache history.

3. The patient had contraindications to additional methods of investigation.

4. The patient refused further examination.

Inclusion criteria for the control group:

1. The patient had no history of stroke, intracranial haemorrhage or other serious neurological and somatic disease, or hereditary connective tissue diseases.

2. The patient had no first degree relatives with intracranial aneurysms, or inherited connective tissue diseases.

3. The patient’s age and sex were matched to patients with SIA.

4. The patient agreed to additional examinations.

We did not perform MR-angiography in control patients since the frequency of aneurysms in the adult population without specific risk factors is only 2-3% [15].

All patients and controls were examined using a specially designed semistructured interview which included detailed information about the history of present and past diseases and the history of their pedigree, results of physical and neurological examinations, physician consultations and treatment. The semistructured interview contained detailed questions about present and past headache disorders allowing diagnosing headaches according to the International Classification of Headache Disorders [14]. The interviews were all performed by the first author (ERL) during examination of patients with SIA at regional neurosurgical center after diagnosis of SIA and before surgical treatment. We asked patients with SIA and controls about headaches during their lifetime and about characteristics of headaches during 1 year before diagnosis of SIA or 1 year before interview in controls. Hypertension was defined as a history of high blood pressure (systolic values ≥140 mm Hg and/or diastolic pressure ≥90 mm Hg) or, if physician observed blood pressure of 140/90 mmHg or above on three consecutive measurements at least six hours apart. Systemic connective tissue dysplasia (CTD) was defined if patient had 3 or more visible markers of connective tissue dysplasia [16]. Smoking was categorized as follows: never smoked, former (regular) cigarette smokers, and current cigarette smoker. Current cigarette smokers were defined as people who reported having smoked ≥100 cigarettes during their lifetime and who still smoked. We also asked about alcohol drinks, how many times per week and quantity in milliliters. Body mass index (BMI) was calculated as the weight in kilograms divided by height in meters squared. 194 patients with SIA (100 females and 94 males) and in 193 age- and sex-matched control patients were asked about occurrence of stroke, SIA, headache and some other disorders in their first degree relatives.

The Medical Ethics Committee of the Urals State Medical Academy approved this study. Informed consent was obtained from all participants.

### Statistical analysis

The differences in mean values or frequencies between patients with SIA and controls were statistically examined by an unpaired *t*-test and chi-square test. Odds ratio (OR) and 95% confidence interval (CI) were estimated using multiple conditional logistic regression models.

## Results

In total we recruited 199 patients with SIA and 194 controls. Before diagnosis, 124 patients with SIA out of 199 had headache (1-year prevalence 62.3%). There were 81 females and 43 males. Age at diagnosis of SIA varied between 14 and 73 years, mean 44.1 years. Patients with migraine were slightly younger than patients with other headaches, mean age 42.7 and 46.2 respectively. The age at the beginning of headache varied between 7 and 62 years (mean 30.2 years). The mean age at the beginning of migraine was significantly lower than in patients with other headaches: 25.7 and 37.5 respectively. The duration of all headaches before diagnosis of SIA varied between 2 months and 43 years (mean 14.8 years). The duration of migraine was longer than of other headaches: 17.4 and 10.5 years respectively. The 1-year prevalence of headaches in controls was 32.5% (63 patients out of 194, 41 females and 22 males) or two times lower than in patients with SIA. Types of headache and other data are presented in Table 2.

**Table 2:** Types of headaches in patients with SIA compare to controls

**Types of headaches**	**Patients with SIA (n = 199)**	**Controls (n = 194)**	**P**	**OR**	**95% CI**
Migraine without aura (MO)	78 (39.2%)	17 (8.8%)	<0.0001	6.7	3.8 -11.9
Migraine with aura (MA)	2 (1%)	0	>0.05		
Probable migraine (PM)	4 (2%)	0	>0.05		
Tension type headache (TTH)	39 (19.6%)	45 (23.1%)	>0.05		
Cluster headache (CH)	2 (1%)	0	>0.05		
Posttraumatic headaches (PH)	2 (1%)	1 (0.5%)	>0.05		

**Figure 1:**
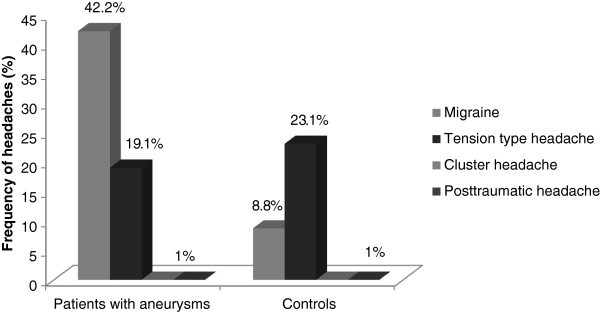
Frequency of headaches in patients with saccular intracranial aneurysms and controls.

Among the different types of headache, only the prevalence of migraine without aura (MO) was significantly higher in patients with SIA than in controls: 39.2% and 8.8% respectively (p < 0.0001, OR 6.7, 95% CI 3.8 -11.9). The frequency of migraine with aura was only 1% and frequency of probable migraine was 2%. In the further analysis of our results we included both migraines with and without aura as well as probable migraine. Therefore 1-year prevalence of migraine was 42.2% in patients with SIA (Figure 1). The migraine headaches in patients with SIA had the typical characteristics of migraine. The typical localization of migraine was fronto-temporal. Their frequency was 1–2 times per months in most cases, only 9 patients had chronic headaches (15 times per month). Four patients had third nerve paresis which developed during severe headache several days before hemorrhages. Many patients told that their headaches were provoked by overheating (bath, sauna) and bending forward. The majority of patients used analgetics 2–4 times per month and 9 patients (10.7%) with frequent headaches used analgetics more than 15 times per month.

**Figure 2:**
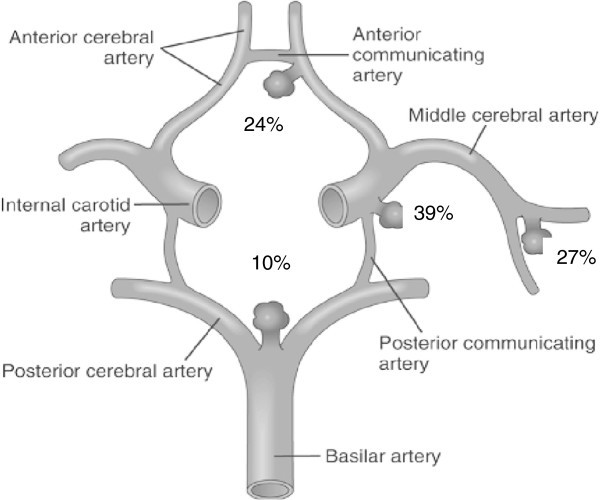
Localization of saccular intracranial aneurysms in 80 patients with migraine.

The localization of SIA in patients with migraine is presented in Figure 2. Aneurysms of the anterior circulation including internal carotid artery (ICA) and posterior communication artery (PCoA), anterior cerebral artery-anterior communicating artery (ACA-ACoA) and middle cerebral artery (MCA) were the most frequent as in patients without migraine. Likewise aneurysms of the posterior circulation (including aneurysms of the basilar artery, vertebral artery and posterior inferior cerebellar artery – PICA) were rare. We compared the laterality of SIA and migraine headache in 63 patients with single aneurysms or with multiple same sided aneurysms (Table 3). The majority - 47 out of 63 (74.6%) patients with single aneurysm had alternating side of headaches but out of those with fixed side (16 patients, 25.4%), 14 always had headache on the same side as the aneurysm and only two had headache on the opposite side.

**Table 3:** Side of SIA and side of migraine with single aneurysms (n = 63)

**Side of SIA/Side of headache**	**Localization of SIA**			
	**AcoA-ACA (n = 18)**	**MCA (n = 16)**	**ICA (n = 23)**	**Posterior circulation (n = 6)**
right/right		1 (6.2%)	4 (17.4%)	1 (16.6%)
left/left	3 (16.6%)	1 (6.2%)	4 (17.4%)	
left/right	1 (5.5%)		1 (4.3%)	
right/alternating sides	8 (44.4%)	8 (50%)	7 (30.4%)	
left/alternating sides	6 (33.3%)	6 (37.5%)	7 (30.4%)	3 (50%)
Basilar artery/alternating sides				2 (33.3%)

In Table 4 we compare the precise localization of SIA and the usual localization of migraine headache. We included in this analysis all single aneurysms and in case of multiple aneurysms – we calculated it separately. 50 out of 80 patients (62.5%) always had same localization (but not fixed laterality) of migraine headache (fronto-temporal, temporal, parietal or occipital) and 30 patients (37.5%) had variable localization of headache. 57 out of 63 patients (90.5%) with single aneurysms had aneurysm of anterior circulation of whom 38 (66.6%) had anterior localization of migraine, 6 patients (9.5%) had aneurysm of the posterior circulation of which 3 (50%) had posterior localization of migraine. 30 patients (37.5%) had variable localization of migraine, among them only 8 (10%) had half-side headache. Among patients who always had the same localization of headache, 27 patients (33.7%) had fronto-temporal migraine headache and 13 patients (16.2%) had fronto-temporal plus other localizations. 13 out of 18 patients (72.2%) with anterior communicating artery aneurysm had fronto-temporal localization of headache.

**Table 4:** Localization of migraine in patients with SIA of different localization (n = 80)

**Localization of migraine**	**Localization of SIA**				
	**AcoA-ACA (n = 18)**	**MCA (n = 16)**	**ICA (n = 23)**	**Posterior circulation (n = 6)**	**Multiple SIA (n = 17)**
**Always same localization of migraine headache**					
Patient uncertain		1 (6.2%)	2 (8.7%)		1 (5.8%)
Temporal	2 (11.1%)	3 (18.7%)	6 (26.1%)	2 (33.3%)	3 (17.6%)
Fronto-temporal	13 (72.2%)	4 (25%)	5 (21.7%)		5 (29.4%)
Occipital		1 (6.2%)	1 (4.3)		
Parietal					1 (5.8%)
**Variable localization of migraine headache**					
Temporal + parietal				1 (16.6%)	2 (11.7%)
Fronto-temporal + occipital	1 (5.5%)	2 (12.5%)			1 (5.8%)
Fronto-temporal + half-side		1 (6.2%)	1 (4.3%)		
Fronto-temporal + parietal		1 (6.2%)	2 (8.7%)		2 (11.7%)
Fronto-temporal + Parietal + occipital	1 (5.5.%)		1 (4.3%)		
Temporal + parietal + occipital + half-side			1 (4.3%)	2 (33.3%)	
Temporal + occipital			4 (17.4%)	1 (16.6%)	1 (5.8%)
Temporal + occipital + half-side	1 (5.5%)				1 (5.8%)
Parietal + occipital		1 (6.2%)			
Parietal + occipital + half-side		1 (6.2%)			
Parietal + half-side		1 (6.2%)			

To reveal factors which might influence the prevalence of migraine in patients with SIA, we compared patients with migraine and patients without migraine (Table 5). We included in this analysis all single aneurysms and, in case of multiple SIA, only the aneurysm causing haemorrhage. The frequency of multiple aneurysms was not significantly different between patients with migraine (21.2%) and without migraine (15.9%). Arterial hypertension (p = 0.007), headaches in first degree relatives (p = 0.007), female gender (p < 0.001) and presence of smoking in females (p = 0.004) were positively associated with the presence of migraine in patients with SIA. Aneurysms of posterior circulation prevailed in patients with migraine (p = 0.05). All other factors including mean age, size, side of SIA, presence of SIA and stroke in first degree relatives, increased BMI and presence of systemic connective tissue abnormalities were not associated with migraine.

**Table 5:** Factors that might influence the frequency of migraine in patients with aneurysms

**Factors**	**Patients with migraine (n = 80)**	**Patients without migraine (n = 119)**	**P**	**OR**	**95% CI**
mean age	42.7	43.8			
Male	20 (25%)	76 (63.9%)	<0.0001	0.19	0.10-0.35
Female	60 (75%)	43 (36.1%)	<0.0001	5.3	2.8-9.9
Smoking	32 (40%)	69 (57.9%)	0.01	0.48	0.27-0.86
Male	18 (22.5%)	63 (52.9%)	<0.0001	0.26	0.14-0.49
Female	14 (17.5%)	6 (5.0%)	0.004	3.99	1.5 – 10.9
AH	55 (68.7%)	59 (49.6%)	0.007	2.2	1.2-4.0
multiple SIA	17 (21.2%)	19 (15.9%)	0.34		
large SIA (11-25 mm)	4 (5%)	7 (5.8%)	0.78		
giant SIA (>25 mm)	6 (7.5%)	5 (4.2%)	0.32		
normal size SIA (5-10 mm)	66 (82.5%)	98 (82.3%)	0.97		
small SIA (<5 mm)	4 (5%)	9 (7.6%)	0.47		
stroke in relatives	24 (30%)	26 (21.8%)	0.19		
SIA in relatives	5 (6.2%)	5 (4.2%)	0.51		
Headaches in relatives	55 (68.7%)	59 (49.6%)	0.007	2.2	1.2-4.0
systemic CTD	51 (63.7%)	73 (61.3%)	0.73		
BMI >25	46 (57.5%)	66 (55.5%)	0.77		
BMI <25	34 (42.5%)	53 (44.5%)	0.77		
Localization of SIA					
ACoA-ACA	19 (23.7%)	55 (46.2%)	0.001	0.36	0.19-0.68
MCA	22 (27.5%)	28 (23.5%)	0.52		
ICA (+PCoA)	31 (38.7%)	32 (26.9%)	0.08		
posterior circulation (vertebral, basilar, PICA)	8 (10%)	4 (3.4%)	0.05	3.19	0.93-10.9
right	40 (50%)	56 (47.1%)	0.68		
left	38 (47.5%)	61 (51.2%)	0.60		
Basilar	2 (2.5%)	2 (1.7%)	0.68		

## Discussion

The main result of this large case control study was that 1-year prevalence of migraine without aura (MO) was significantly higher in patients with saccular intracranial aneurysms (42.2%) than in controls (8.8%) and also significantly higher than in the general population of Russia (20.8%) [17]. Migraine with aura was not increased although all patients were interviewed in detail about aura symptoms. 1-year prevalence of tension type headaches was almost the same in patients with SIA (19.6%) and controls (23.1%) but it was below the prevalence of TTH in the general population of Russia (30.8%) (17). We did not interview about rare headaches which may be the reason for our low prevalence of TTH in SIA and controls. The frequency of migraine in our controls was lower than in the general population of Russia, possibly because blood donors could perhaps be healthier than the general population.

### Previous studies of unruptured intracranial aneurysms and headache

The literature before the use of the International Classification of Headache Disorders (ICHD-1) [14,18,19] is impossible to judge today. Only very few more recent studies are available. Raps et al. described headaches in 18 out of 111 patients (16.2%) with unruptured SIA [20]. Most other publications were case reports about patients with SIA who suffered migraine [21-25]. The low frequency of headache in previous studies can be explained by a lack of purpose to study headache and no use of a detailed interview about headaches. An interesting study was performed by Schwedt et al. [12]. They analyzed headache patterns prior to and following treatment of unruptured intracranial aneurysms in 44 patients and identified factors associated with different headache outcomes. The majority of patients (approximately 2/3) with pretreatment headaches had substantial reductions in headache frequency during the 6 months following treatment. Potential predictors for the absence of headache improvement following aneurysm treatment were: having migraine prior to treatment, having more severe headaches prior to treatment, stent-assisted coiling and higher pretreatment trait anxiety. Baron EP et al. (2011) found that stent-assisted coiling may provoke development of post-procedural headache [26].

### Strengths and weaknesses of the present study

In relation to the previous literature the present study has many advantages. It has a very large study population. Its cases and comparable controls were interviewed prospectively and in parallel, interviews were conducted entirely by one of the authors (E.R.L.) – a neurologist with an interest in headache and subarachnoid hemorrhage – and the patient material is very large. Random statistical variation is thus highly unlikely to explain the results. All patients and controls originated from one defined geographical region of Russia and the great majority were ethnic Russians – thus limiting variability. The semi structured interview was designed specifically for the present study and included all items necessary to classify patients according to the International Classification of Headache disorders second edition. Since such a neurologist conducted interview is the gold standard of headache diagnosis, it was not validated. Finally, extensive information about the aneurysms and their rupture allowed a correlation between the location of headache and the location and type of aneurysm. A weakness of the present study is the lack of information about rare and mild headaches. The fact that patients were interviewed after rupture about the headache before rupture could possibly influence the results(missing of very rare headache and sometimes headache characteristics in rare headache) but the most likely effect of the bleed would be a lack of memory about previous headaches, which would probably decrease the prevalence of reported headache. Furthermore it would be impossible to accrue a sufficiently large material of patients with unruptured aneurysms. Patients who were aphasic, confused or with decreased level of consciousness were excluded from the present study. The presence of migraine with aura, 1%, was surprisingly low. However, the one year prevalence in the population is only 4% or 8 out of 200 subjects. We had 2 and this difference could be due to random variation. On balance, the present results are the most reliable data so far about the relationship between headache and unruptured SIA.

### Reasons for increased prevalence of MO in SIA

There are several possible explanations for the increased prevalence of MO in SIA. The patients had a markedly increased prevalence of arterial hypertension (AH) compared to the normal control subjects. However, the relation between AH and migraine is uncertain. Some studies have found an increased prevalence of arterial hypertension in migraine [27] while others studies have shown no association to blood pressure [28] or revealed that only the diastolic blood pressure is elevated in migraine patients [29]. While AH is clearly a risk factor for SIA [30] it is therefore much less clear that it is a risk factor for MO. In our own material, SIA patients with migraine had a significantly higher prevalence of hypertension than SIA patients without migraine. This can, however, explain only a small part of the increased risk of migraine without aura in SIA patients. SIA is associated with an increased prevalence of systemic connective tissue abnormalities [16] but in the present study there was no difference in the prevalence of MO in SIA patients with or without connective tissue abnormalities. Thus, systemic factors can explain only a small part of the increased prevalence of migraine in SIA patients. Aneurysms compressing cranial nerves or other structures are known to cause headache but usually it does not have the characteristics of MO. Furthermore, only 4 patients had compression of the third nerve and only 11 patients had giant aneurysms which were likely to compress intracranial structures. Out of these 15 patients 48% had MO which is similar to the whole material. On balance, neither systemic nor these two local factors explain the observed marked increase in the prevalence of MO.

It seems most likely, therefore, that it is the aneurysm itself which in some way is triggers MO. When headaches were always on the same side, it was on the side of the aneurysm in 14/16 cases. Furthermore, a fronto-temporal localization was most prevalent with aneurysms of the anterior communicating artery and anterior circulation aneurysms were most often associated with anterior migraine pain. There is, thus, some correlation between the localization of the aneurysm and migraine headache. On the other hand, the majority of cases had migraine headache on alternating sides just like in MO patients without SIA. On this basis it seems unlikely that a stimulus from the aneurysm is directly responsible for the migraine pain. More likely, input from perivascular sensory nerve terminals around the aneurysm may be a stimulus that increases sensitization in the central nervous system. This would lower the threshold for migraine to be elicited by a host of other factors, factors that probably are identical to those triggering MO in patients without SIA. Many experimental studies have demonstrated how increased sensory input by a localized stimulus e.g. inflammatory soup on the dura mater [31-33], can induce a wide spread and even contralateral sensitization. Such mechanisms are likely to operate also in patients with SIA and to explain the increased prevalence of otherwise typical attacks of MO in these patients. It would be interesting to follow patients with unruptured aneurysms with repeated quantitative sensory testing in the face and scalp.

One might ask why MO but not MA had increased prevalence. MO has been associated with sensory input from the big arteries at the base of the brain, i.e. where SIA is typically located. MA is believed to be secondary to cortical spreading depression which is more likely to be induced by cortical pathology. These relations have recently been discussed [34].

## Conclusion

In conclusion, the present study demonstrates a markedly increased prevalence of migraine without aura in patients with unruptured saccular intracranial aneurysms. There was a correlation between the localization of aneurysms and the localization of migraine attacks but there were even bigger discrepancies. Systemic factors explained only a small part of the increased prevalence. We suggest that increased sensory input from sensory nerve endings around the aneurysms may sensitize the central nervous system and thus decrease the threshold for developing spontaneous migraine attacks.

## Competing interests

The authors declare that there is no conflict of interest.

## Authors’ contributions

ERL participated in designing the study, did all interviews, and performed statistical analysis. She wrote the first version of the manuscript. NMG carried out the follow-up of these patients and created data base. VPS participated in planning the study and delivered the cases. JO participated in planning and writing of the publication. All authors read and approved the final manuscript.
